# Characterization of Zebrafish Pax1b and Pax9 in Fin Bud Development

**DOI:** 10.1155/2014/309385

**Published:** 2014-08-13

**Authors:** Xuemei Chen, Huizhe Huang, Hua Wang, Fengjin Guo, Xiaogang Du, Linqiang Ma, Liang Zhao, Zhuma Pan, Haibo Gui, Taixian Yuan, Xin Liu, Lin Song, Yiquan Wang, Junling He, Han Lei, Rui Gao

**Affiliations:** ^1^Faculty of Basic Medical Sciences, Chongqing Medical University, Medical College Road 1, Chongqing 400016, China; ^2^Emergency Department, The First Affiliated Hospital of Chongqing Medical University, Youyi Road 1, Chongqing 400042, China; ^3^School of Life Sciences, Xiamen University, South Xiang'an Road, Xiamen 361102, China; ^4^Department of Nephrology, The First Affiliated Hospital of Chongqing Medical University, Youyi Road 1, Chongqing 400042, China; ^5^Department of Cardiology, The First Affiliated Hospital of Chongqing Medical University, Youyi Road 1, Chongqing 400042, China

## Abstract

Both Pax1 and Pax9 belong to the important paired box gene family (PAX), which mainly participates in animal development and sclerotome differentiation. To date, the precise molecular mechanism and related signaling pathway of Pax1 remain unclear. In our study, microinjection of morpholino- (MO-) modified antisense oligonucleotides against *pax1b* induced pectoral fin bud defects. Furthermore, we demonstrate that the phenotypes caused by the knockdown of Pax1b in zebrafish could not be phenocopied by *pax9* MO and could not be rescued by either Pax1a or Pax9 overexpression. We further find that Pax1b affects the expression of *col2a1*, Uncx4.1, Noggin3, and *aggrecan*, confirming the role of Pax1b in chondrocyte differentiation and bone maturation. Moreover, we identify an interaction between PAX1 and FOXO1 and find that the interaction was enhanced under hypoxia stress. Together, this evidence for cell death caused by *pax1b* knockdown provides new insight into the role of the Pax protein family in cell fate determination and tissue specification.

## 1. Introduction

The Pax protein family, consisting of numerous transcription factors with a paired box domain containing 128 amino acids, plays a central role in embryonic patterning and organ differentiation [1]. In vertebrates, *Pax* genes are divided into four subfamilies according to their structures. The *Pax1/9* subfamily participates in the formation of skeletal muscle and sclerotome differentiation [2, 3].

In most vertebrates, Pax1 and Pax9 have similar expression patterns and functions. For example, the expression of both chicken* PAX1* and* PAX9 *genes was the strongest in undifferentiated cells of precartilage condensations or at the margins of differentiated cartilages and was absent from cartilage itself [4]. Both induce chondrogenic differentiation in the sclerotome via targeting *Nkx3.2* [5]. Murine *PAX1* and *PAX9* have overlapping expression profiles and respond to fibroblast growth factor (FGF) and hedgehog (HH) signaling during the progression of limb bud formation [6]. More interestingly, there are four kinds of spontaneous* Pax1* mutant mice (*Pax1* ^*un*^ *, Pax1* ^*un-ex*^ *, Pax1* ^*un-i*^, and* Pax1* ^*Un-s*^) which show different phenotypes [7]. It has been reported that* PAX1* is a candidate gene in vertebral malformations and congenital scoliosis from the study of clinical genetics and the mouse mutant* undulated* [8, 9]. Using the teleost medaka, a closely related species to zebrafish, Japanese scientists determined the similarity of* pax1* and* pax9* expression patterns in the sclerotome and pharyngeal pouch. MO knockdown of either Pax1 or Pax9 causes defects in the neural arch and scoliosis and double knockdown revealed that Pax1 and Pax9 function synergistically in sclerotome development [10].

However, the expression patterns of* pax1b* and* pax9* in zebrafish are quite different.* pax1b* is a maternally expressed gene and is zygotically expressed in the pharyngeal pouches, fin bud, and notochord and weakly expressed in the dorsal aorta and axial vein at 48 hpf [11], while* pax9* is expressed after segmentation, primarily in part of the somites and branchial arches and not in the fin bud (ZFIN). These differences in their expression patterns suggest divergent functions in transcriptional activity and cell differentiation between Pax1b and Pax9 in zebrafish. To address whether the functions of Pax1b and Pax9 have distinct roles in zebrafish embryonic development, we designed two morpholinos (MOs) against* pax1b* and* pax9* to study their mechanism of action.

FOXO1, a member of the Forkhead family proteins of the O subclass, is not only one of the most critical regulators of cell death [12], but also an early molecular regulator during mesenchymal cell differentiation into osteoblasts. In mouse embryos, the expression of* FoxO1* is higher in skeletal tissues, and *FoxO1* silencing has a drastic impact on skeletogenesis and craniofacial development [13]. Gene fusions involving* PAX3/7* and *FOXO1* in alveolar rhabdomyosarcoma have been reported [14]; however, the interaction between PAX1 and the FOXO family has not yet been described. In this research, we studied the relationship between PAX1 and FOXO1 to determine whether FOXO1 participates in the developmental processes regulated by Pax1.

## 2. Materials and Methods

### 2.1. Fish Maintenance and Embryo Collection

Zebrafish (*Danio rerio*), AB strain, were kept at 28.5°C under a light and dark cycle of 14 and 10 hours, respectively. Embryos were collected and staged as described [15].

### 2.2. Plasmid Construction

The* pax1b* cDNA sequence was deposited in GenBank with an accession number of XM_695785. The full coding sequence of* pax1b* was amplified from cDNAs derived from 24 hpf embryos with a forward primer (zp1F: 5′-atgcaaatggatcagacgtac-3′) and a reverse primer (zp1R: 5′-ttatgagtctgagagtccatg-3′) and subcloned into pXT7 and pBlueScript to generate vectors for synthesizing mRNA and antisense RNA probes* in vitro*, respectively. Zebrafish* pax9* and amphioxus* pax1/9 *were subcloned using the same strategy as* pax1b* and the primers were as follows: zp9F (5′-atggagccagcctttgg-3′), zp9R (5′-tcatagagctgaagccaccag-3′), ap1/9F (5′-atgatgaatatggagcaaacatttg-3′), and ap1/9R (5′-ttatgaggaggaagcggatg-3′). Expression plasmids were all subcloned into pCMV5 vector with various tags. Template for PCR was cDNA from different species including human, mouse, and zebrafish.

### 2.3. Reverse Transcription-PCR

To quantify* nkx3.2, col2a1*, and* aggrecan* transcripts in embryos, injected embryos were digested at 24 hpf or 48 hpf. First strand cDNAs synthesized from total RNA (Trizol from Takara) were used as templates with the SuperScript Kit (Invitrogen). Specific primers with the sequences listed in Supplemental Table  1 (see Supplementary Material available online at http://dx.doi.org/10.1155/2014/309385) were used to amplify markers [16, 17]. TE buffer was used as negative control. For qPCR assays, fold change for each group of embryos was determined using the delta-delta Ct method. Data were normalized to the control embryos. Quantified mRNA levels were normalized to *β*-*actin* and are presented relative to control embryos.

### 2.4. RNA Synthesis, Whole-Mount* In Situ* Hybridization

Capped mRNAs were synthesized using T7 Cap Scribe (Roche) according to the manufacturer's instructions. For preparation of digoxigenin-labeled antisense probe, plasmid containing* pax1b* cDNA was linearized with* Kpn*I.* In situ* hybridizations were performed as previously described [18].

### 2.5. Morpholinos and Microinjection

Four morpholino oligonucleotides were synthesized by Gene Tools (*pax1b*-MO1: 5′-CATTTGCATTGTGATATTTCCCTAT-3′, positioned from 176 to 200 in the ENSDART00000132835 sequence;* pax1b*-MO2: 5′-CCCGTGTCTCCCGCTAAAGACTGCC-3′, positioned from 84 to 108 in ENSDART00000132835; zebrafish* pax9*-MO1: 5′-CAAAGGCTGGCTCCATTGCGTTTAG-3′, positioned from 136 to 160 in the U40931.1 sequence; and zebrafish* pax9*-MO2: 5′-GCTGGTAATTATTGCACCGAAGCCG-3′, positioned from 47 to 71 in the U40931.1 sequence). The sequence of control MO is 5′-CCTCTTACCTCAGTTACAATTTATA-3′. All MOs were dissolved in nuclease-free water to make a 20 *μ*g/*μ*L stock. Western blots and RT-PCR assays were used to check MO efficiency. All morphants were injected using a 1 : 1 mixture of the two MOs. mRNAs and morpholino oligonucleotides were injected into the yolk of fertilized eggs at the single-cell stage [16].

### 2.6. Cell Culture and Cell Death Assay

Mammalian cells were grown in DMEM (GIBCO) supplemented with 10% fetal calf serum (Hyclone). In the DNA damage induced cell death assay, U2OS cells were exposed to lethal treatments (80 J/m^2^ UV or 2.5 *μ*M doxorubicin) and were kept in culture medium for 8 h before Hoechst staining. The positive cell numbers in 10 random sweeps were summed, and an error bar was calculated from 3 independent replications in each panel [19].* pax1b* DNA induction was mediated by the IRES-TOMATO lentivirus system.

### 2.7. Western Blot, Immunoprecipitation, and Immunofluorescence Staining

36 hpf embryos and 293FT cells were lysed with lysis buffer [18]. The total lysis was mixed with an equal volume of 2× SDS sample buffer and was analyzed by Western blotting. Antibodies used are the following: rabbit polyclonal antibody to Pax1 (83312 from Abcam), Uncx4.1 (ARP47548 from Aviva Systems Biology), Noggin3 (16054 from Abcam), and FOXO1 (sc-11350 from Santa Cruz) and mouse monoclonal antibody to Flag (F1804 from Sigma) or Myc (M4439 from Sigma). For immunoprecipitation, anti-Flag M2 affinity gel was purchased from Sigma. For immunofluorescence staining, 24–36 h after transfection with or without hypoxia stimulation, HeLa cells grown on coverslips were fixed with 4% formaldehyde for 20 min at room temperature, followed by 0.5% Triton X-100 treatment for 5 min and 3% BSA blocking. The cells were then incubated with corresponding primary and secondary antibodies along with DAPI staining for visualization of nuclei. Fluorescence images were acquired with a Nikon microscope. Fluorescent secondary antibodies, Alexa Fluor 546 (A10040), and FITC-Goat anti-mouse antibody (62-6511) were purchased from Invitrogen.

### 2.8. Hypoxia Treatment

293FT and HeLa cells were treated with CoCl_2_, a well-known hypoxia mimetic agent [20] at different concentrations (2, 20, 200, and 400 *μ*mol/L) for 14 h.

### 2.9. Statistical Analysis

Data are presented as means ± SE. Differences between treatment groups were analyzed using ANOVA. Differences were considered significant at the *P* < 0.05 level.

### 2.10. Ethics Statement

Our experiments were conducted with the permission of the ethics committee of Chongqing Medical University.

## 3. Results

### 3.1. Pax1b Is Required for Zebrafish Morphogenesis and Embryonic Development

**Figure 1:**
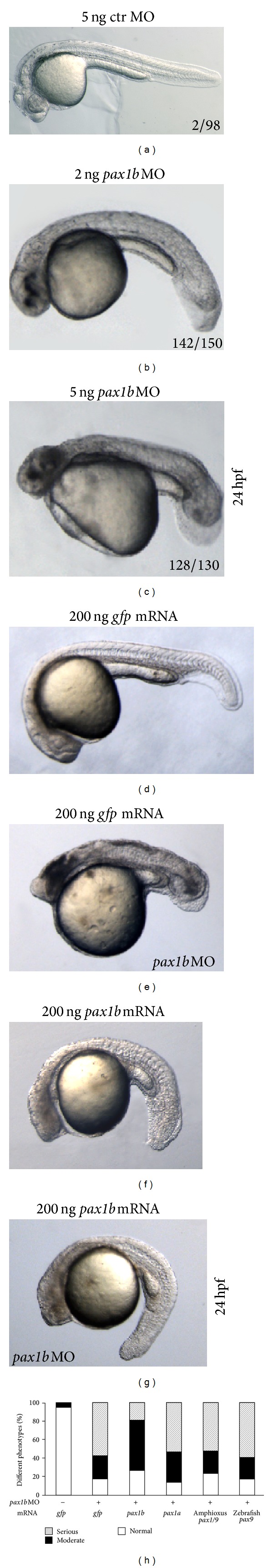
Phenotype caused by* pax1b* knockdown. In order to analyze the function of* pax1b* during embryogenesis, we injected* pax1b*-specific morpholinos into fertilized eggs to block production of functional Pax1b protein. (a) Embryo injected with 5 ng control morpholino. ((b)-(c)) The phenotypes of* pax1b* morphants caused by 2 ng or 5 ng injection doses. ((d)–(g)) The phenotypes caused by coinjection of 200 ng* pax1b* or* gfp* mRNA with 5 ng* pax1b *MO or control MO.* pax1b* mRNA could partially rescue the defective fin bud phenotype of the* pax1b* morphant (g);* gfp* mRNA failed to rescue defective fin bud phenotype of the* pax1b* morphant (e). (h) Statistical analysis of phenotypes caused by coinjection of different mRNAs with 5 ng* pax1b* MO. 150 embryos were calculated. The amount of mRNA injected for every embryo is as follows: 200 ng* gfp* mRNA, 200 ng* pax1b* mRNA, 200 ng* pax1a* mRNA, 150 ng amphioxus* pax1/9* mRNA, and 200 ng zebrafish* pax9* mRNA. All embryos were observed at 24 hpf. zpax1b: zebrafish pax1b, zpax1a: zebrafish pax1a, zpax9: zebrafish pax9. hpf: hours post-fertilization. gfp: green fluorescence protein. ctr: control.

In the context of bone mineralization and sclerotome differentiation, few studies to date have examined Pax1b function in zebrafish. We designed two MOs against* pax1b* to block its translation (Figure S1). Western blot assays and RT-PCR showed that the bands in the 2nd and 3rd lanes had reduced signals with respect to the control lane (Figure S2A), confirming the efficiency of* pax1b* MOs on protein and RNA levels, respectively. Zebrafish embryos injected with 2 ng* pax1b* MO showed small eyes as well as a curved axis and tail, while 5 ng* pax1b* MO caused more severe phenotypes: head atrophy and a shorter body axis, indicating that the* pax1b* MO functions in a dose-dependent manner (Figures 1(b) and 1(c)).

To test the specificity of* pax1b* MOs, we carried out coinjection of* pax1b* mRNA and* pax1b *MO and found that 200 ng of* pax1b* mRNA could rescue* pax1b* morphants to normal axial length, while* gfp *mRNA could not rescue the axis defects at any concentration (Figures 1(d)–1(g)). Interestingly,* pax1a, *the closest homologue of* pax1b,* could not rescue* pax1b* morphants in our experiments, neither could amphioxus* pax1/9 *nor could zebrafish* pax9*, the other member of the* pax1/9* subfamily (Figure 1(h)). These results indicated that, compared with* pax1a* and zebrafish* pax9, pax1b *plays different roles in early embryonic development and teleost Pax family members have more diverse and complex functions than previously shown.

### 3.2. Pax9 Inhibition Causes a Tail Defect

**Figure 2:**
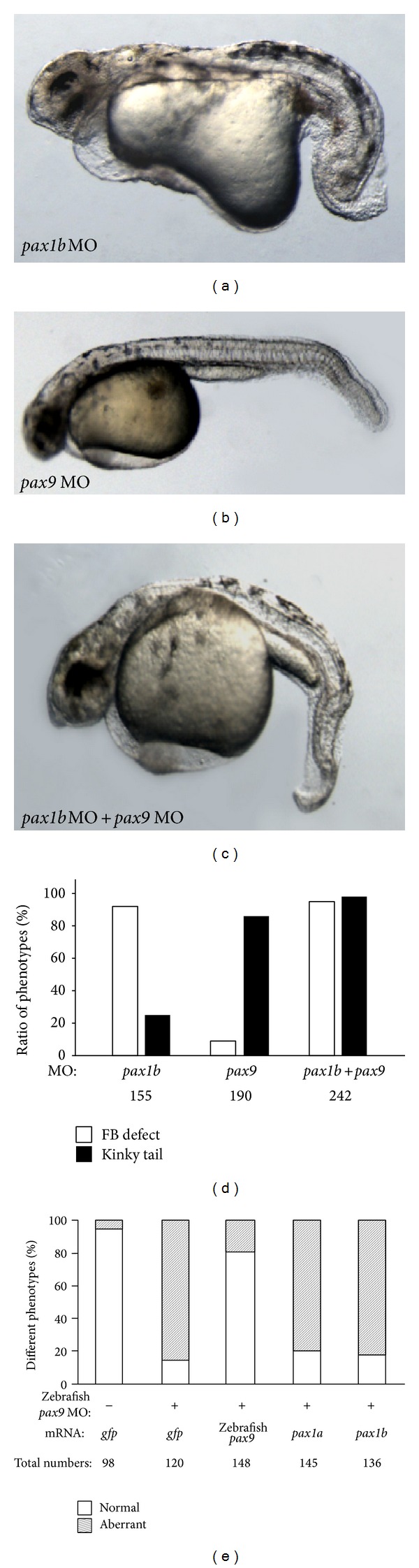
Fin bud and tail defects caused by injection of* pax1b* MO and/or zebrafish* pax9* MO. (a) Fin bud and axis defects caused by injection of 5 ng* pax1b* MO. (b) Tail defect caused by injection of 5 ng zebrafish* pax9* MO. (c) Fin bud and tail defects caused by coinjection of 2.5 ng* pax1b* MO and 2.5 ng zebrafish* pax9* MO. (d) Statistics of phenotype ratios. White columns indicate the ratio of fin bud defect; shaded columns indicate the ratio of kinky tail. (e) Statistical analysis of phenotypes caused by coinjection of different mRNAs with 5 ng zebrafish* pax9* MO. The amount of mRNA injected for every embryo is as follows: 200 ng* gfp* mRNA, 200 ng* pax1b* mRNA, 200 ng* pax1a* mRNA, and 200 ng zebrafish* pax9* mRNA. All embryos were observed at 36 hpf.

Two MOs against zebrafish* pax9 *were designed (Figure S1). Due to the lack of a Pax9 antibody, we verified zebrafish* pax9* MO efficiency using its target *nkx3.2* (Figure S2B). As expected, the knockdown of zebrafish* pax9* downregulated* nkx3.2* transcription, confirming the efficiency of* pax9 *MOs. Zebrafish* pax9* morphants have different phenotypes than* pax1b* morphants.* pax1b* morphants showed short body axis and a fin bud defect; however, zebrafish* pax9* morphants only showed a kinked tail. Coinjection of* pax1b *andzebrafish* pax9 *MO showed all of the defects mentioned above (Figures 2(a)–2(d)). The tail defect in zebrafish* pax9* morphants could be rescued only by zebrafish* pax9* mRNA, while the aberrant phenotype could not be rescued by mRNA of members of the same subfamily,* pax1a *and* pax1b*. These results further confirm that* pax1* and* pax9* have unique functions in zebrafish embryo development.

### 3.3. Loss of Function of Zebrafish* pax1b* Causes Fin Bud Defects

**Figure 3:**
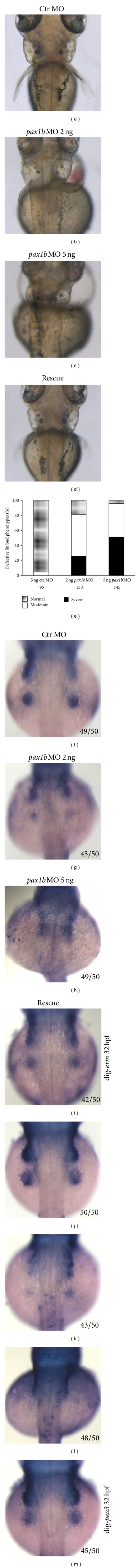
Fin bud defects caused by* pax1b* knockdown. ((a)–(d)) Dorsal view of the region near the fin bud with orientation of head towards the top. (a) Control embryos. ((b)-(c))* pax1b* morphants. (d) Rescue embryos: coinjection with 5 ng* pax1b* MO and 200 ng* pax1b* mRNA. (e) Statistical analysis of fin bud defects caused by injection of control MO or* pax1b* MO. Total numbers of injected embryos are labeled. ((f)–(m)) Expression pattern of* erm *and* pea3* in control embryos,* pax1b* morphants, and coinjected embryos. Numbers of defective embryos and total numbers of stained embryos are labeled in the bottoms. The embryonic stage is 72 hpf in panels (a)–(e) and 32 hpf in panels (f)–(m). All the embryos are viewed from the dorsal side with head towards the top.

Compared with the control group, single-cell stage injection of 2 ng* pax1b* MO caused moderate defects, with smaller and asymmetric pectoral fin in 55% of embryos, and severe defects including the almost complete lack of fin buds in 27% of embryos. Embryos injected with 5 ng* pax1b* MO had more serious phenotypes: fin buds were nearly abolished in about 52%* pax1b* morphants (Figures 3(a)–3(c) and 3(e)). Coinjection of* pax1b* MO and* pax1b* mRNA rescued the aberrant phenotypes, confirming the specificity of the* pax1b* MO (Figure 3(d)). In order to further characterize the observed phenotypes, we evaluated the expression of the pectoral fin markers* erm* and* pea3*, as means to assess defects in fin bud development. In* pax1b* morphants at 28 hpf, the expression of* erm* and* pea3 *was dramatically reduced. Coinjection of* pax1b* MO and* pax1b* mRNA rescued the defects (Figures 3(f)–3(m)). These data confirm that* pax1b* plays a vital role in zebrafish fin bud development.

### 3.4. Pax1b Controls Bone Maturation

**Figure 4:**
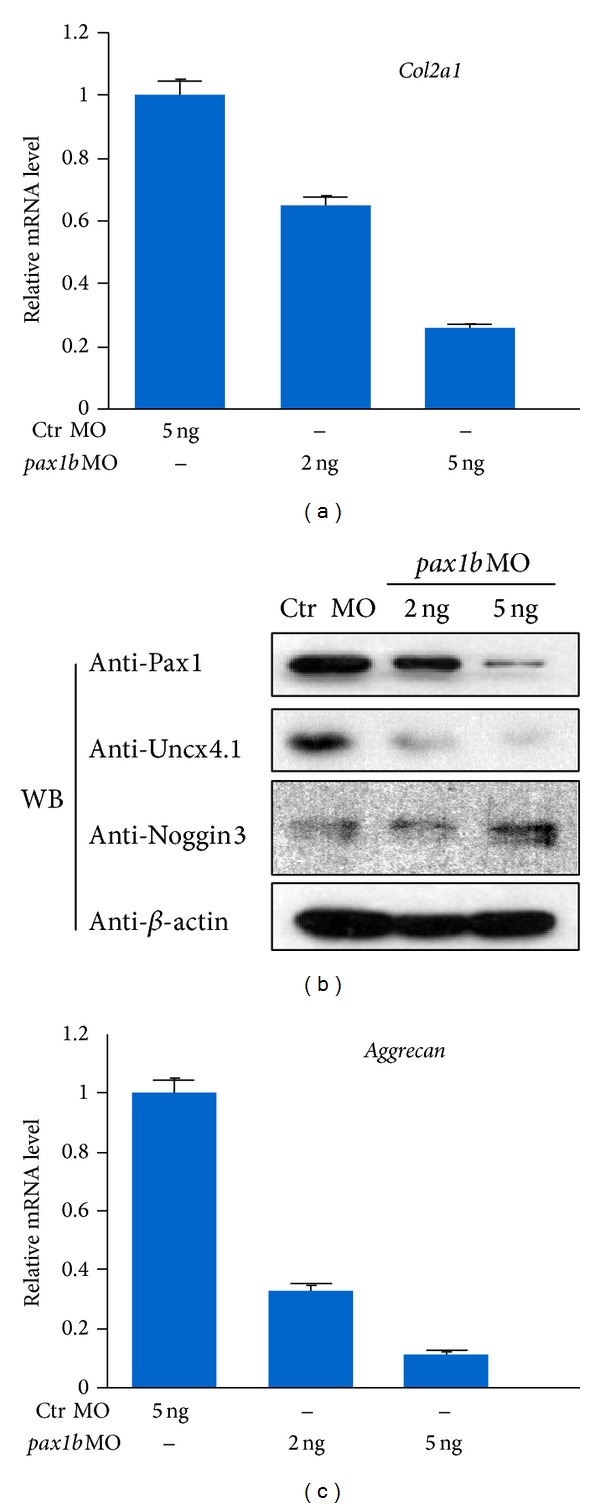
Molecular mechanism of sclerotome development mediated by Pax1b. (a) The relative expression level of zebrafish collagen type II (*col2a1*) monitored by qPCR. (b) Sclerotome differentiation analyzed by molecular markers Uncx4.1 and Noggin3 by Western blot. (c) The relative expression level of zebrafish *aggrecan* monitored by qPCR. The fold change for each group was determined using the delta-delta Ct method. Quantified mRNA levels were normalized to *β*-*actin* and are presented relative to control embryos. 50 embryos at the 48 hpf stage were used in each group, performed in triplicate.

Further investigation at the molecular level found that collagen type II (*col2a1*), a chondrocyte differentiation marker, was downregulated in* pax1b* morphants (Figure 4(a)), suggesting that chondrocytes differentiation was affected in* pax1b* morphants. Using polyclonal antibodies against Noggin3 and Uncx4.1, we found that the protein level of Uncx4.1 was downregulated, while Noggin3 was upregulated in* pax1b* morphants (Figure 4(b)). Due to a lack of available antibody against Aggrecan, we detected its transcript and found a significant reduction in* pax1b* morphants (Figure 4(c)). These results suggested that* pax1b* correlates with the progression of bone maturation.

### 3.5. Forced Expression of Pax1b Decreases Cell Death Potential on Physiological Stress

**Figure 5:**
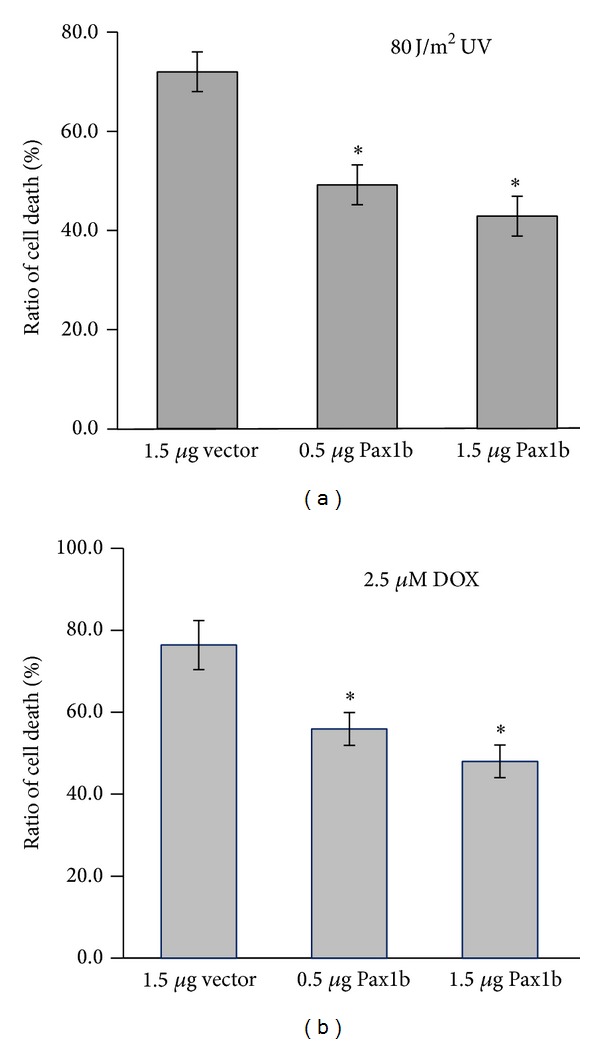
Forced expression of Pax1 decreases cell death potential on physiological stress. U2OS cells were exposed to 80 J/m^2^ UV (a) or 2.5 *μ*M doxorubicin (b) for 8 h. Hoechst-positive cells were counted and subjected to statistical analysis using Student's *t*-tests. All* pax1b* DNA induction was mediated by the IRES-TOMATO lentivirus system. Data are presented as means ± SE from three independent experiments. ∗*P* < 0.05, compared with vector.

The obvious fin bud defects in* pax1b* morphants led us to investigate whether Pax1b affected cell death in an overexpression system. A range of biological stressors or DNA damage can induce cell death. In control cell culture, UV treatment with 80 J m^−2^ dose induced 72.0% cell death in the U2OS cell line, but transfection of 0.5 *μ*g or 1.5 *μ*g* Pax1b* DNA reduced this rate to 49.2% and 42.9%, respectively (Figure 5(a)). Consistently, 2.5 *μ*M doxorubicin caused 76.4% U2OS cell death; transfection of 0.5 *μ*g or 1.5 *μ*g Pax1b DNA decreased this ratio to 55.9% and 48%, respectively (Figure 5(b)). Thus, Pax1b serves as a cell death inhibitory molecule, and its knockdown might increase apoptosis or other types of cell death.

### 3.6. PAX1 Interacts with FOXO1

**Figure 6:**
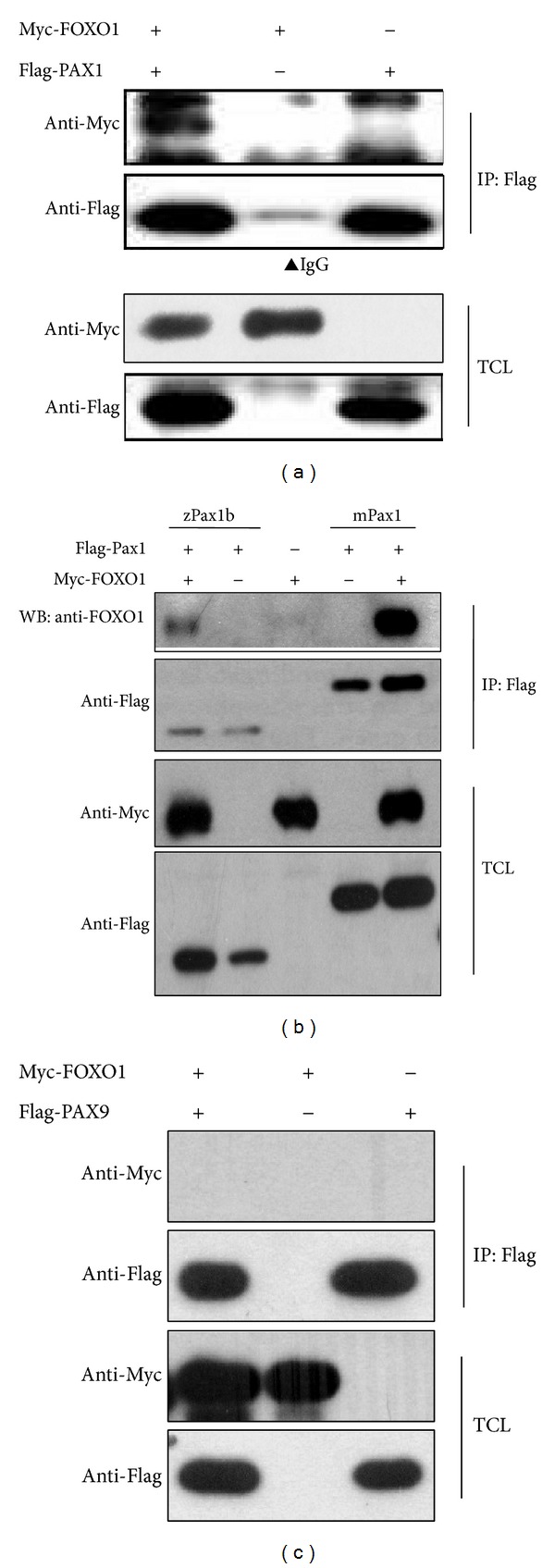
The interaction between PAX1 and FOXO1. (a) FOXO1 is a new PAX1-interacting protein. HEK293FT cells were transfected with Myc-tagged FOXO1 and Flag-tagged PAX1 cloned from human cDNA. Cells were harvested for immunoprecipitation with anti-Flag affinity resin and immunoblotted with the indicated antibodies. (b) The interaction of Pax1 and FOXO1 was conserved. Myc-tagged FOXO1 was immunoprecipitated by Flag-tagged Pax1b or mouse Pax1. (c) PAX9 did not interact with FOXO1 in HEK293FT cells. zPax1b: zebrafish Pax1b; mPax1: mouse Pax1. TCL: total cell lysate. IP: immunoprecipitation.

We studied the relationship between PAX1 and FOXO1 using immunoprecipitation assays. Results showed that PAX1 interacts with FOXO1 in HEK293FT cells and that the interaction is conserved in different species including human, mouse, and zebrafish (Figures 6(a) and 6(b)). However, there was no interaction between PAX9 and FOXO1 (Figure 6(c)).

**Figure 7:**
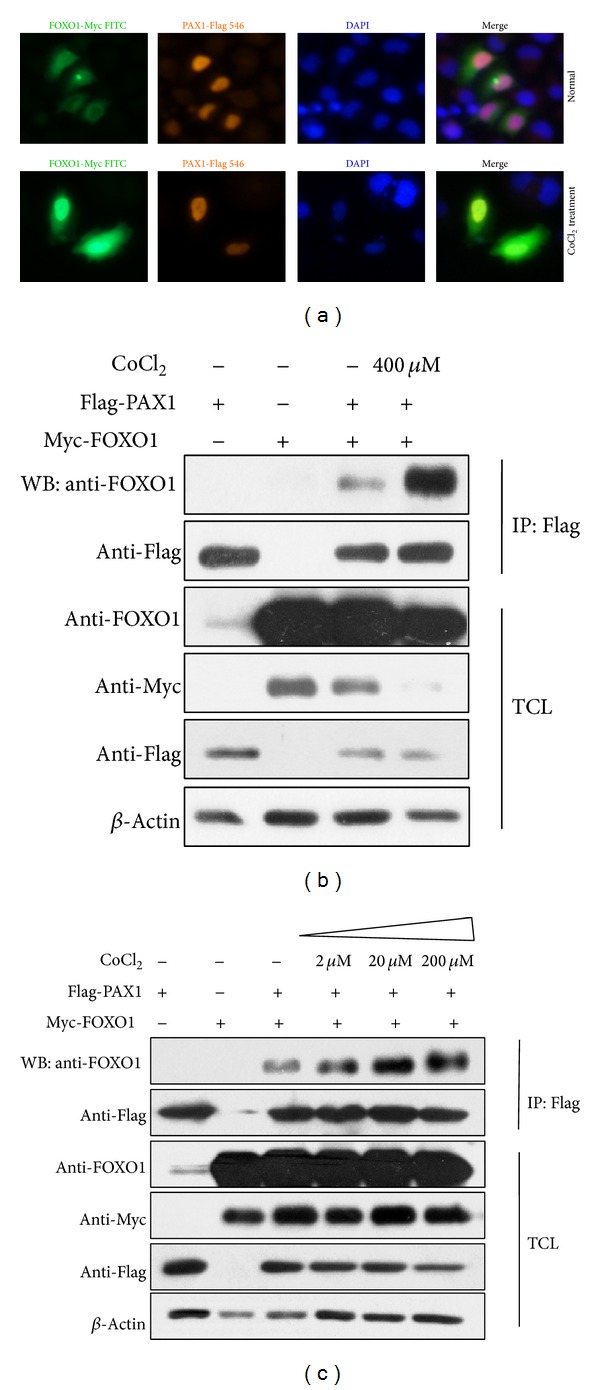
The interaction between PAX1 and FOXO1 is enhanced under hypoxia stress. (a) Colocalization of PAX1 and FOXO1 in HeLa cells. Myc-FOXO1 is distributed in the cytoplasm and nucleus while Flag-PAX1 is located in the nucleus only. The colocalization of PAX1 and FOXO1 was increased after treatment with 20 *μ*M CoCl_2_ for 14 h. Myc-FOXO1 was cotransfected with Flag-PAX1 into HeLa cells. 36 h after transfection, cells were subjected to immunostaining using anti-Myc antibody, anti-Flag antibody, and DAPI and observed by microscopy. ((b)-(c)) 2 *μ*g Flag-tagged PAX1 and/or 0.5 *μ*g Myc-tagged FOXO1 were transfected into HEK293FT cells, respectively. Semiendogenous Co-IP revealed that the interaction of PAX1 and FOXO1 was strengthened when stimulated with CoCl_2_ and the enhancement occurred in a dose-dependent manner.

We next tested the subcellular localization of PAX1 and FOXO1. Immunofluorescence assays revealed that PAX1 was only located at the nucleus while FOXO1 was distributed in both the cytoplasm and nucleus. Moreover, the colocalization of PAX1 and FOXO1 increased when stimulated with CoCl_2_ (Figure 7(a)). Further coimmunoprecipitation analysis showed that hypoxia stress enhanced the interaction between PAX1 and FOXO1 in a dose-dependent manner (Figures 7(b) and 7(c)). Taken together, these data provide evidence that Pax1 might participate in fin bud development together with FOXO1.

## 4. Discussion

The PAX protein family was first identified almost thirty years ago [21]. We used MEGA4.0.2 software to do a phylogenetic analysis and found that there are high identities among* Homo sapiens* Pax1,* Mus musculus* Pax1,* Xenopus* Pax1,* Danio rerio* Pax1,* Danio rerio* Pax9, and* Branchiostoma* Pax1/9 (Figure S3). The amino acid alignment performed by DNAssist software indicated that the amino acid sequence is highly conserved among* Branchiostoma* Pax1/9,* Danio rerio* Pax1a, and* Danio rerio* Pax1b as well as* Danio rerio* Pax9 (Figure S4). The knockout of* Pax1* in mouse produced malformed sternum and scapula [7]. In this report, we used zebrafish as an animal model to investigate the biological functions of Pax1b and Pax9, demonstrating that* pax1b* morphants display serious defects in fin buds and the axis which is different than* pax9* morphants. Pax1b overexpression rescued the morphants to a moderate phenotype, whereas Pax1a or zebrafish Pax9 could not rescue* pax1b* morphants. On the other hand, several reports have revealed differences between* undulated* mutations of* Pax1* and its knockout model, proving the haploinsufficiency of Pax1 and redundancy of Pax9 [7, 22]. Moreover, it has been reported that the loss of Pax9 function in the vertebral column in Pax9^lacZ^ mutant mice might be rescued by Pax1 and another report showed that Pax9 might partially substitute for Pax1 [7, 23]. Our results suggest that Pax1b cannot rescue zebrafish* pax9* morphants nor can zebrafish Pax9 rescue* pax1b* morphants which suggests that the divergence of two subfamily members has biological significance and is responsible for the different physiological or environmental stresses in the evolutionary process.

The development of fin buds is related to the formation of cartilage and chondral ossification. The chondrogenic anlage is the main component of the fin bud mesenchyme in zebrafish [24]. Recent research reported that* pax1b* knockdown leads to hypoplasia in pharyngeal cartilage [11]. We found that loss of function of* pax1b* in zebrafish downregulated the expression of* col2a1,* a chondrocyte differentiation marker. In the perichondral or endochondral ossification through the cartilage anlagen, Uncx4.1 and Aggrecan served as positive regulators, and Noggin3 is regarded as negative regulator in this process [25–27]. Our results indicated that the expression of Uncx4.1, Noggin3, and Aggrecan is disturbed in* pax1b* morphants. All of these results confirm that Pax1b plays vital roles in fin bud development.

The size of an organ is largely determined by the number of cells it contains and cell death is an essential aspect in this process [28]. The phenotype of fin bud defects in* pax1b* morphants might have a close relationship with cell death. Previous studies have illustrated that Pax3 and Pax7 are associated with cell survival in numerous cancer cell lines and silencing of* pax2* promotes renal carcinoma apoptosis [29, 30]. Our original study shows that knockdown of* pax1b* induced cell death in the specific tissue of zebrafish embryos and Pax1b overexpression decreased stress-induced apoptosis in the U2OS cell line. FOXOs not only promote mammalian cell survival by inducing cell cycle arrest and quiescence in response to oxidative stress, but also regulate longevity in model organisms [31]. FOXO1 can be phosphorylated by JNK or Mst1 proteins, which phosphorylate FOXO1 under conditions of oxidative stress. This phosphorylation causes the translocation of FOXO1 from the cytoplasm to the nucleus [12]. It has been reported that the transcription of FoxO3, another member in the FoxO subfamily, is induced by hypoxia and the increased expression of FoxO3 results in enhanced cellular survival by attenuating HIF-induced apoptosis [32]. We supposed that FOXO1 might play a role in resistance to hypoxic stress during development together with PAX1. In this study, we demonstrate for the first time that PAX1 interacts with FOXO1 and that this interaction is strengthened under hypoxia stress. We postulate that fin bud malformation in* pax1b* morphants is caused by cell death via FOXO1 signaling. The downstream events remain unclear, and much more work is needed in the future to address the exact mechanism.

## 5. Conclusions

In summary, we have discovered that* pax1b* plays a pivotal role in zebrafish fin bud development. Overexpression of* pax1b* can relieve cell death induced by stress. Furthermore, we found an interaction between PAX1 and FOXO1 for the first time, an interaction enhanced under hypoxia stress. Together, the evidence for cell death caused by* pax1b* knockdown provides new insights into the role of the Pax protein family in cell fate determination and tissue specification.

## Supplementary Material

Figure S1: Schematic diagram of pax1b and zebrafish pax9 translation blockers.
Figure S2: pax1b and zebrafish pax9 morpholino efficiency.
Figure S3: Phylogenetic analysis of Pax1a, Pax1b and Pax9 proteins from following species: Danio rerio, Xenopus tropicalis, Xenopus laevis, Mus musculus, Homo sapiens, Branchiostoma belcheri.
Figure S4: Amino acid sequence alignment done with DNAssist software.
Supplemental Table 1: Primer sequences used in RT-PCR.
