# Supplementary material for: Characterization of Zebrafish Pax1b and Pax9 in Fin Bud Development
Source: Biomed Res Int. 2014 Aug 13;2014:309385. doi: 10.1155/2014/309385 (PMC4147360; doi:10.1155/2014/309385)
Supplement: Supplementary file 1 — Figure S1: Schematic diagram of pax1b and zebrafish pax9 translation blockers. Figure S2: pax1b and zebrafish pax9 morpholino efficiency. Figure S3: Phylogenetic analysis of Pax1a, Pax1b and Pax9 proteins from following species: Danio rerio, Xenopus tropicalis, Xenopus laevis, Mus musculus, Homo sapiens, Branchiostoma belcheri. Figure S4: Amino acid sequence alignment done with DNAssist software. Supplemental Table 1: Primer sequences used in RT-PCR. [file 309385.f1.pdf]

Supporting Information

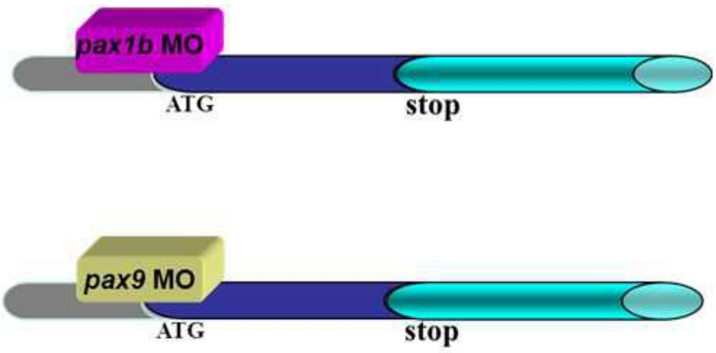

Figure S1. Schematic diagram of *pax1b* and zebrafish *pax9* translation blockers. The three-dimensional cubes represent two independent MOs and are not to scale.

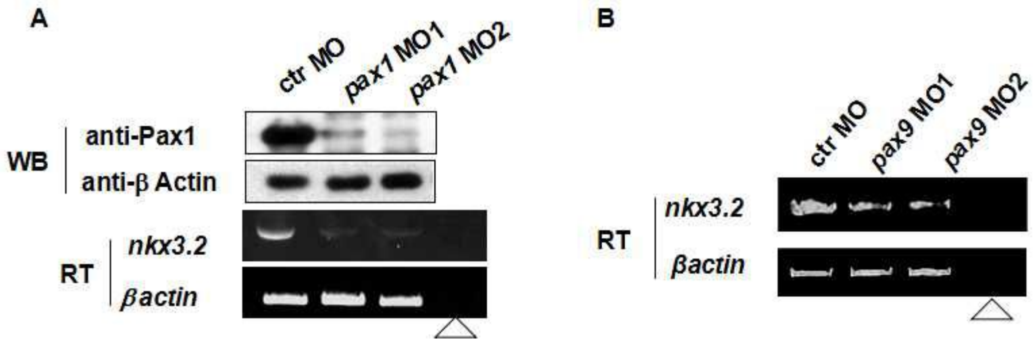

Figure S2. *pax1b* and zebrafish *pax9* morpholino efficiency. (A) Western blot and RT-PCR analysis demonstrated that *pax1b* morpholino disrupted the production of PAX1 and its target *nkx3.2* cDNA, respectively. (B) zebrafish *pax9* morpholino reduced the production of its target *nkx3.2* cDNA assessed by RT-PCR. The triangles indicate the negative control, using TE buffer without template.

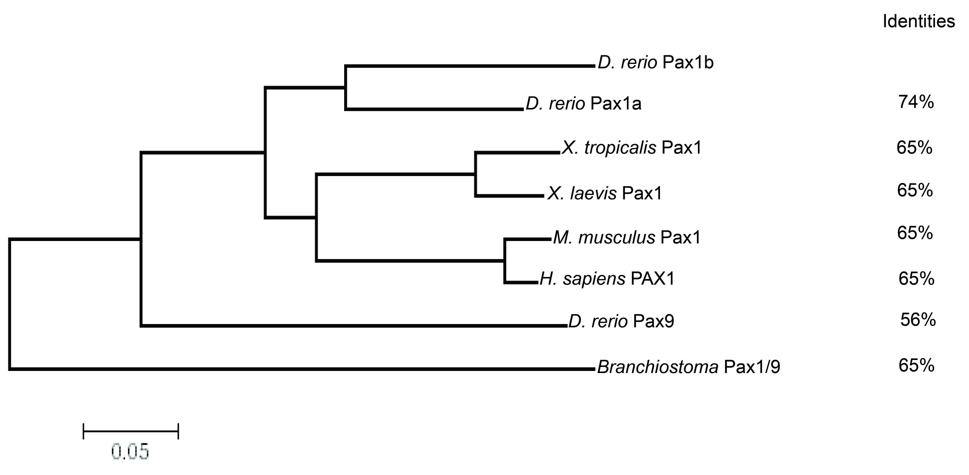

Figure S3. Phylogenetic analysis of Pax1a, Pax1b and Pax9 proteins from following species: *Danio rerio, Xenopus tropicalis, Xenopus laevis, Mus musculus, Homo sapiens, Branchiostoma belcheri.*

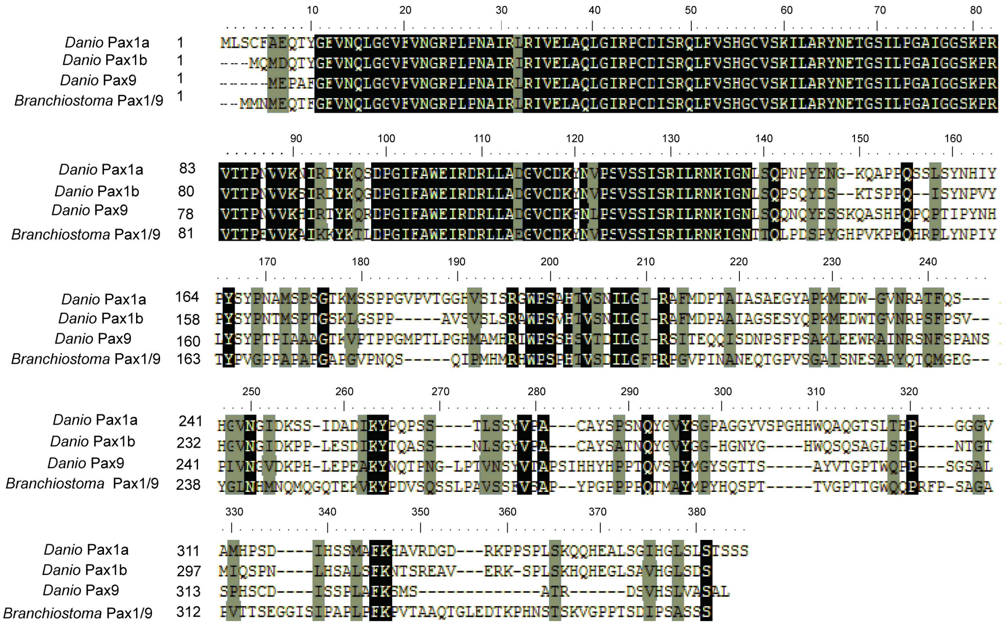

Figure S4. Amino acid sequence alignment done with DNAssist software. Black boxes represent identical amino acid residues, and gray boxes represent conserved amino acid residues.

Supplemental Table 1. Primer sequences used in RT-PCR.

Primer Name	Primer Sequences	Fragment Length
nkx3.2 forward	atggctgtgcgcagtaactc	738
nkx3.2 reverse	tcactggtttccagagcaggc	
aggrecan forward	gggggtccagaggagcaagc	519
aggrecan reverse	ttagagttgtttctccaatgc	
col2a1a forward	ccattaggaccaccgggtgc	156
col2a1a reverse	aaatcctgacctggtgttgg	
actin forward	atggatgatgaaattgccgcac	475
actin reverse	accatcaccagagtccatcacc	
